# NUF2 overexpression predicts poor outcomes in multiple myeloma

**DOI:** 10.1016/j.gendis.2024.101268

**Published:** 2024-03-19

**Authors:** Shanshan Zhang, Li Zhang, Linjing Cai, Huan Chen, Yuqi Wang, Youhai Yuan, Hanzhen Zhang, Xiaolei Wei

**Affiliations:** a Department of Hematology, Nanfang Hospital, Southern Medical University; Clinical Medical Research Center of Hematological Disease of Guangdong Province, Guangzhou, Guangdong 510515, China; b Department of Blood Transfusion, The First Affiliated Hospital of Guangzhou Medical University, Guangzhou, Guangdong 510120, China

Multiple myeloma (MM) is the second most common hematologic malignancy and is characterized by the expansion of clonally plasma cells and related organ or tissue damage.^1^ The clinical behavior, response, and survival of MM are heterogeneous, and much of this variability may be driven by acquired genetic factors, including primary immunoglobulin translocations, hyperdiploid, and additional copy number gains and losses.^2^ The abnormalities of chromosome 1q (+1q) were found in about 30%–40% of patients with newly diagnosed MM and at a higher proportion at the refractory/relapse stage.^3^ The gain/amplification of 1q (1q gain/amp) has been reported to relate to poor outcomes due to the dysregulation of lots of genes located in 1q including CKS1B, ANP32E, and ADAR1.^3^ However, the other prognostic genes on 1q and the underlying mechanisms remain elusive.

To identify the key gene irregularities in MM, we conducted a differential expression analysis in MM patients with and without 1q gain/amp, utilizing the dataset GSE4581. A total of 959 differentially expressed genes were identified, with 541 up-regulated and 418 down-regulated (absolute logFC ≥ 0.5 and adjusted *P*-value < 0.01) (Fig. S1A). The 165 candidate genes were obtained by overlapping these up-regulated genes with 2242 genes located on 1q (Fig. S1B). To further identify key prognostic genes located on 1q, we applied LASSO Cox regression analysis and screened out nine prognosis-related genes (*P* < 0.001; ANP32E, ARPC5, CDC42BPA, CHD1L, COA6, ISG20L2, NUF2, TAGLN2, and UBE2T) for further investigation (Fig. 1A). Of the nine genes, CDC42BPA was not detected and eight other genes were further validated in a single-cell RNA sequencing dataset. We observed that NUF2 was mainly and uniquely overexpressed in relapsed/refractory MM patients (Fig. 1B). Ndc80 kinetochore complex component (NUF2), located in chromosome 1q23.3, is a crucial substance that stabilizes spindle microtubule-kinetochore attachment throughout the metaphase of cell division.^4^ Mounting evidence has shown that NUF2 is up-regulated in multiple human cancers and involved in tumorigenesis and immune infiltration.^5^ However, the prognostic value of NUF2 in MM has not been studied. Survival analysis revealed that high NUF2 expression implied inferior overall survival in MM patients from dataset GSE4581 (*P* < 0.001; Fig. 1C). Besides, the adverse prognostic significance of NUF2 overexpression remains apparent in MM patients regardless of the presence or absence of chromosome 1q abnormality (*P* < 0.001; Fig. S1C).

**Figure 1:**
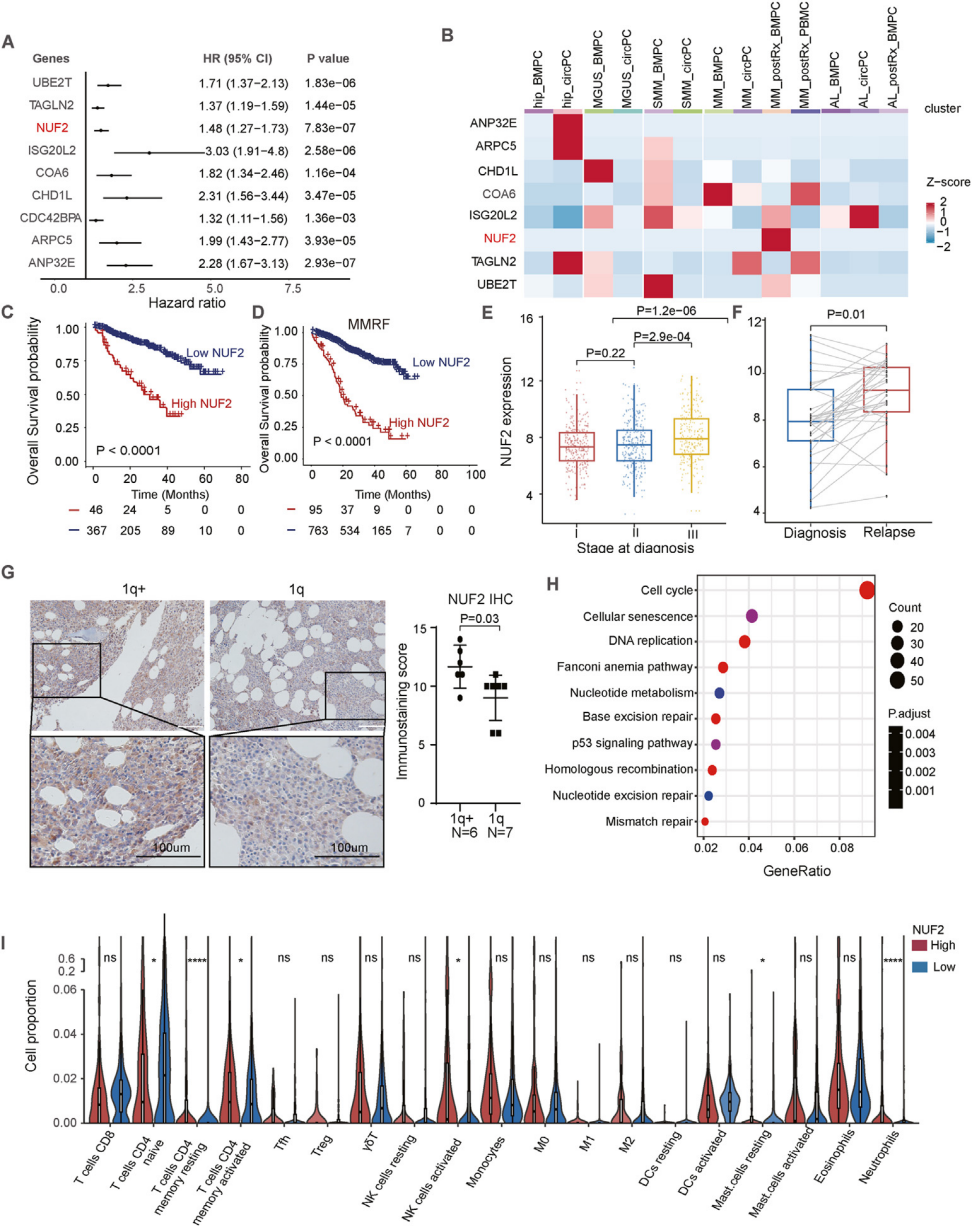
NUF2 overexpression predicts poor outcomes in multiple myeloma (MM). **(A)** Forest plot of the univariate Cox regression analysis of overall survival of nine candidate genes. **(B)** The expression profiles of eight candidate genes in a single-cell RNA sequencing dataset. **(C)** The Kaplan–Meier curves comparing overall survival between individuals with high NUF2 expression (red) and those with low NUF2 expression (blue). **(D)** Survival curves of overall survival from dataset MMRF. **(E)** The expression of NUF2 in different stages of MM. **(F)** The relation of NUF2 expression with diagnosis and relapse. **(G)** Immunostaining of NUF2 expression in MM patients with 1q gain/amp (1q+; *n* = 6) versus MM patients without 1q gain/amp (1q; *n* = 7). The scatter plot shows the immunostaining scores of NUF2 with the mean and 95% confidence interval (CI) indicated by a bar. **(H)** The top 10 KEGG pathways in the enrichment analysis of the NUF2 co-expressed genes. **(I)** The violin plots comparing the proportions of immune cells between the high- and low-NUF2 expression. The red bars represent the low-risk groups, and the blue bars represent the high-risk groups. Statistical significance was evaluated by Wilcox tests. ns, *P* > 0.05; ∗*P* ≤ 0.05, ∗∗*P* ≤ 0.01, ∗∗∗*P* ≤ 0.001, ∗∗∗∗*P* ≤ 0.0001. The *P* values in (C, D) were calculated by the Log-rank test, the *P* values in (E, F) were calculated by the *t*-test, and the *P* values in (G) were calculated by the Wilcox test.

To further confirm the prognostic value of NUF2 in MM patients, we performed survival analysis independently in datasets GSE24080 and MMRF. Consistent with the result in GSE4581, NUF2 overexpression predicted shorter event-free survival and overall survival in MM patients (*P* < 0.001; Fig. 1D; Fig. S1D, E). Multivariate survival analysis showed that NUF2 overexpression, independent of age, creatinine, lactate dehydrogenase, albumin, and cytogenetic abnormalities, predicted shorter event-free survival (hazard ratio = 2.99; 95% confidence interval = 1.93–4.63; *P* < 0.001; Fig. S1F) and overall survival (hazard ratio = 2.72; 95% confidence interval = 1.61–4.60; *P* < 0.001; Fig. S1G). Next, we constructed a prognostic nomogram that integrated NUF2 and all other significant clinical features (Fig. S1H). The area under the receiver operator characteristic curve of 1-, 3-, and 5-year overall survival predictions were 0.806, 0.826, and 0.822, respectively (Fig. S1I).

We employed different datasets with clinical information available to further explore the relation of NUF2 expression with known high-risk clinical characteristics in MM patients. In the dataset GSE24080 with 558 MM patients included, the clinical characteristics including age (*P* = 0.933), gender (*P* = 0.861), different serotype (*P* = 0.165), and β2-microglobulin (*P* = 0.367) showed no significant differences in MM patients with and without NUF2 overexpression (Fig. S1J). However, MM patients with NUF2 overexpression tended to exhibit elevated lactate dehydrogenase levels (*P* < 0.001), higher creatinine concentrations (*P* = 0.009), and more cytogenetic abnormalities (*P* < 0.001) (Fig. S1K). The data from the MMRF dataset showed stage III MM patients showed a significantly increased NUF2 expression compared with stage I/II MM patients (*P* < 0.001; Fig. 1E). The data from GSE39754 showed that NUF2 expression was significantly increased in relapsed MM patients (*P* < 0.001; Fig. 1F). We also explored the impact of NUF2 expression on treatment response in GSE39754 but no relation was found between NUF2 expression and overall response rate (*P* = 0.882; Fig. S1L). To confirm the overexpression of NUF2 in the tumor cells from MM patients with 1q gain/amp, we conducted immunohistochemistry and observed a higher immunostaining score for NUF2 in the bone marrow of MM patients with 1q gain/amp (1q+; *n* = 6) compared with patients without 1q gain/amp (1q; *n* = 7) (*P* = 0.03; Fig. 1G).

The above results indicated that NUF2 overexpression in MM patients could serve as a biomarker for higher tumor burden, increased risk of relapse, and poorer outcomes. To explore the potential molecular mechanisms underlying the NUF2 overexpression that contribute to MM progression, KEGG and GO enrichment analyses were performed in multiple datasets to identify the enriched specific signaling pathways differentially expressed in high NUF2 MM patients. The results from the dataset GSE24080 showed that the KEGG signaling pathways enriched consisted of cell cycle, cellular senescence, and DNA replication (Fig. 1H). In addition, GO analysis showed that the candidate genes were significantly enriched in the regulation of chromosome segregation, nuclear division, and DNA-templated replication in terms of biological process. The chromosomal region, condensed chromosome, and chromosome centromeric region in terms of cellular composition were significantly enriched. The catalytic activity acting on DNA, DNA helicase activity, and single-strand DNA helicase activity as an acceptor in terms of molecular function were significantly involved by candidate genes (Fig. S1M). Similar results were also found in the datasets GSE2658 and GSE4581 (data not shown). We also constructed a protein–protein interaction network of these candidate genes visualized using Cytoscape software (Fig. S1N). Cell cycle, cellular senescence, and DNA replication related genes were downloaded from KEGG. Among them, we selected the top 20 genes ranked by *P* value and constructed protein–protein interaction network and correlated heatmap to NUF2 (Fig. S1O). We applied CIBERSORT to deconvolute the infiltration levels of 22 immune cell types in MM patients from the dataset MMRF according to NUF2 expression (Fig. S1P). We observed higher levels of tumor infiltrations of CD4^+^ T cell memory resting, CD4^+^ T cell memory activated, natural killer cell activated, mast cell resting, and neutrophils in MM patients with NUF2 overexpression (Fig. 1I). These results demonstrated that NUF2 may contribute to MM carcinogenesis by regulation of multiple signaling pathways involved in the process of DNA replication, cell cycle, and tumor immune micro-environment.

In summary, this is the first study to find that NUF2 overexpression implied poor outcomes in MM. Moreover, our findings suggest that NUF2 may act as an oncogene by regulating the cell cycle and the tumor-infiltrating immune cells. Further molecular biology experiments are required to comprehensively investigate the biological functions of NUF2 in MM.

## Author contributions

WXL designed the study. ZSS, CLJ, CH, WYQ, YYH, and ZHZ collected and analyzed the data. WXL and ZL drafted the manuscript. All authors read and approved the final manuscript.

## Funding

This work was supported by Natural Science Foundation of Guangdong, China (No. 2024A04J5216), the Technology Project of Guangzhou City (Guangdong, China) (No. 202102020937), and the 10.13039/501100010112Outstanding Youth Development Scheme of Nanfang Hospital, 10.13039/501100010096Southern Medical University (Guangdong, China) (No. 2019J011).

## Conflict of interests

The authors declare no conflict of interests.

## Appendix A Supplementary data

Multimedia component 1 Multimedia component 1

Figure S1 Identification and validation of the prognostic value of CRIP1 in multiple myeloma (MM). **(A)** Volcano plot of differentially expressed genes in MM patients with and without 1q gain/amp. **(B)** The Venn diagram illustrating the overlap between the up-regulated genes in Chr1q (+) patients and the genes located on Chr1q. **(C)** The Kaplan–Meier curves comparing the overall survival of MM patients grouped by NUF2 expression and 1q gain/amp. **(D)** Survival curves of event-free survival from dataset GSE24080. **(E)** Survival curves of overall survival from dataset GSE24080. **(F)** Multivariate Cox proportional hazards regression for event-free survival from dataset GSE24080. **(G)** Multivariate Cox proportional hazards regression for overall survival from dataset GSE24080. **(H)** A prognostic nomogram that integrated NUF2 expression and other prognostic factors for overall survival in MM from GSE24080. **(I)** A calibration curve at 1-, 3-, and 5-year. **(J)** The heatmap showing the main clinical characteristics and *P* values of MM patients grouped by high and low NUF2 expression. **(K)** The boxplots displaying the relations of NUF2 expression to main clinical parameters. **(L)** The relation of NUF2 expression to treatment response. **(M)** The GO functional enrichment analysis of the NUF2 co-expressed genes. **(N)** The top 20 genes of the cell cycle, cellular senescence, and DNA replication correlated with NUF2 in the protein–protein interaction network. **(O)** Correlation between NUF2 and main candidate genes enriched. The correlation coefficients were calculated by Pearson correlation analysis. ∗*P* ≤ 0.05, ∗∗*P* ≤ 0.01, ∗∗∗*P* ≤ 0.001. **(P)** The landscape of the composition of the 22 immune cells in each sample, and the samples are in ascending order of NUF2 expression along the *x*-axis. The *P* values in (C–E) were calculated by the Log-rank test, the *P* values in (F–H) were calculated by the autoReg R package, and the *P* value in (*L*) was calculated by the chi-square test. Figure S1

## References

[1] Cowan A.J., Green D.J., Kwok M. (2022). Diagnosis and management of multiple myeloma: a review. JAMA.

[2] Kumar S.K., Rajkumar S.V. (2018). The multiple myelomas - current concepts in cytogenetic classification and therapy. Nat Rev Clin Oncol.

[3] Hanamura I. (2021). Gain/amplification of chromosome arm 1q21 in multiple myeloma. Cancers.

[4] Ciferri C., Pasqualato S., Screpanti E. (2008). Implications for kinetochore-microtubule attachment from the structure of an engineered Ndc80 complex. Cell.

[5] Zheng B., Wang S., Yuan X., Zhang J., Shen Z., Ge C. (2023). NUF_2_ is correlated with a poor prognosis and immune infiltration in clear cell renal cell carcinoma. BMC Urol.

