# Supplementary material for: NUF2 overexpression predicts poor outcomes in multiple myeloma
Source: Genes Dis. 2024 Mar 19;12(1):101268. doi: 10.1016/j.gendis.2024.101268 (PMC11550750; doi:10.1016/j.gendis.2024.101268)
Supplement: Multimedia component 1 [file mmc1.docx]

**Materials and methods**

**Sample collection**

Thirteen bone marrow biopsy samples were collected from MM patients collected from the Nanfang Hospital. All participants signed informed consent and the study was approved by the Ethics Committee of the Nanfang Hospital.

**Data sources**

The gene expression matrix and clinical data of patients with MM were obtained from the National Center for Biotechnology Information (NCBI) Gene Expression Omnibus (GEO) including GSE24080 (n=559), GSE39754 (n=136) and GSE4581(n=414). The TCGA MM RNA sequencing dataset (MMRF-CoMMpass) which contains 787 cases with MM was drawn from The Cancer Genome Atlas as a training dataset in our study.

**Identification of key gene on chromosome 1q**

The differentially expressed genes (DEGs) were calculated by comparing with MM patients with or without 1q gain/amp using the R package ‘limma’(version 3.54.0). The upregulated genes overlapped with the genes located in chromosome 1q were included in univariate and multivariate Cox regression to identify the potential genes related to prognosis. Finally, these candidate genes were confirmed at single-cell resolution.

**IHC**

IHC staining and immunostaining score of NUF2 was performed as previously described [1]. In brief, after the sections of paraffin-embedded tissue were deparaffinized and rehydrated, the tissues were incubated using the antibody NUF2 (1:200; No. 15731-1-AP; Proteintech, USA). The intensity of NUF2 was graded as follows: 0, negative; 1, weak; 2, moderate; and 3, strong. The percentage of NUF2-positive cells were evaluated as follows: 0, negative; 1, 0-25%; 2, 26-50%; 3, 51-75%; and 4, 76-100%. The stained sections were evaluated by two pathologists. The immunostaining score was determined by the multiplication of the intensity and positive percentage.

**Gene set variation analysis (GSVA) and functional enrichment analyses**

GSVA is a non-parametric and unsupervised method for evaluating the transcriptomic gene set enrichment. Gene sets were downloaded from the Molecular Signatures Database (version 7.0) and each gene set was comprehensively scored by the GSVA algorithm, and the potential differences in biological functions between the high- and low-risk groups were explored. Gene Ontology (GO) and Kyoto Encyclopedia of Genes and Genomes (KEGG) analysis of candidate genes were carried out by enrichGO and enrichKEGG functions of clusterProfiler package. GO terms and KEGG pathways with P and Q values < 0.05 were considered statistically significant.

**Immune Infiltration Analysis**

The R package ‘CIBERSORT’(version 0.1.0) was employed to evaluate the relative proportions of 22 infiltrating immune cell types in MM. Spearman correlation analysis was performed to evaluate the potential correlation between the gene signature and immune cells infiltration.

**Statistical Analysis**

R software (version 4.2.2) was used for all statistical analysis. Overall survival (OS) and event-free survival (EFS) were analyzed by the Kaplan-Meier method using the R package ‘survival’(version 3.4.0). Multivariate Cox proportional hazards regression was used to identify independent prognostic factors. All P values were two-sided and the significance was defined as p<0.05.

**References**

[1] Lin, J., Chen, X., Yu, H., Min, S., Chen, Y., Li, Z., & Xie, X. (2022). NUF2 Drives Clear Cell Renal Cell Carcinoma by Activating HMGA2 Transcription through KDM2A-mediated H3K36me2 Demethylation. International journal of biological sciences, 18(9), 3621–3635.
