# Supplementary figures and images for: NUF2 overexpression predicts poor outcomes in multiple myeloma
Source: Genes Dis. 2024 Mar 19;12(1):101268. doi: 10.1016/j.gendis.2024.101268 (PMC11550750; doi:10.1016/j.gendis.2024.101268)

Figure S1

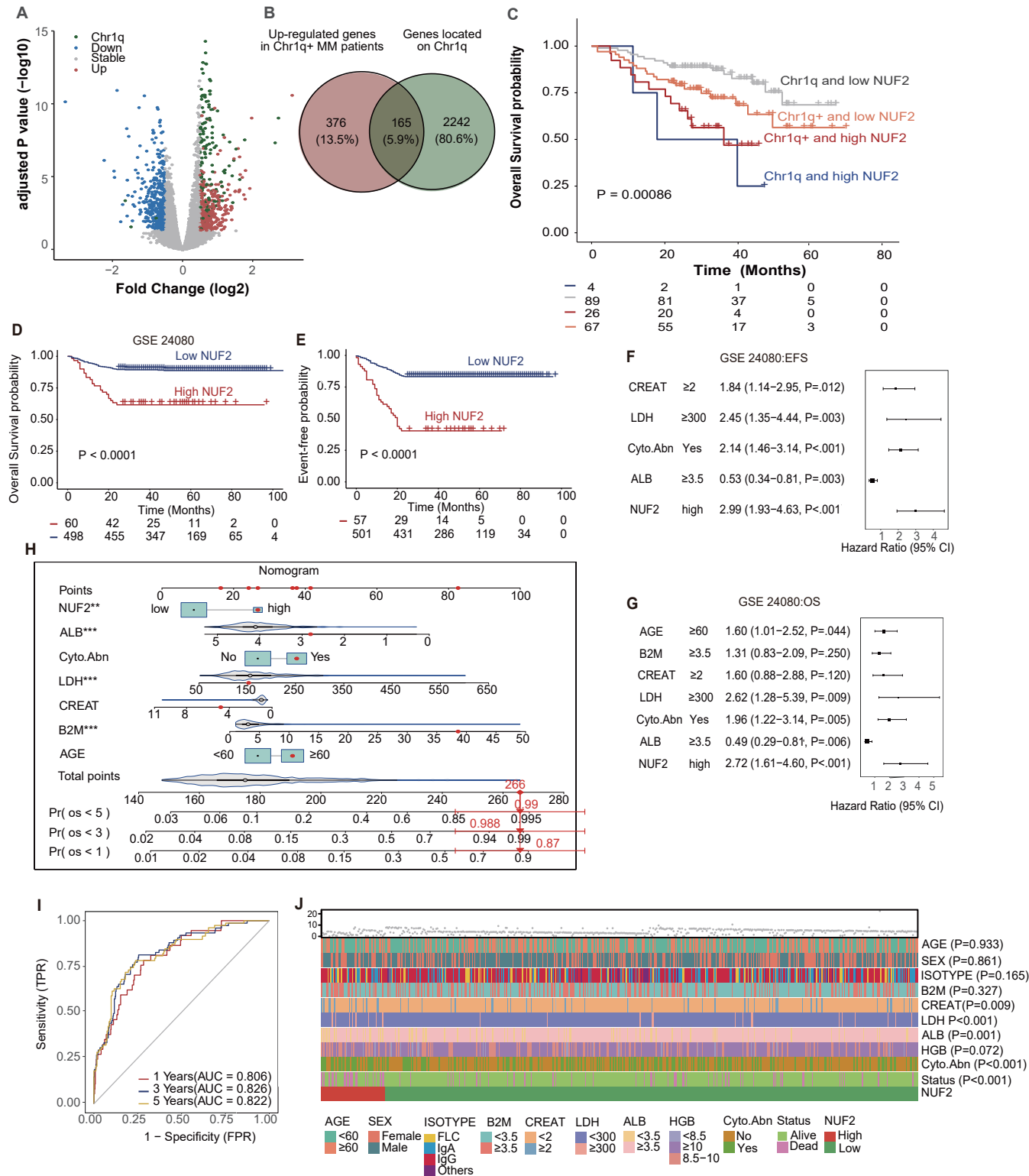

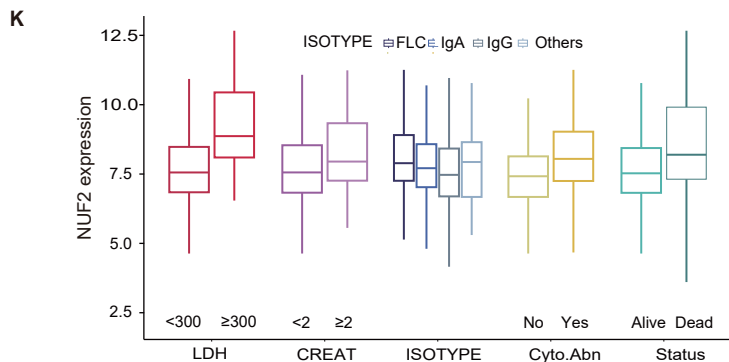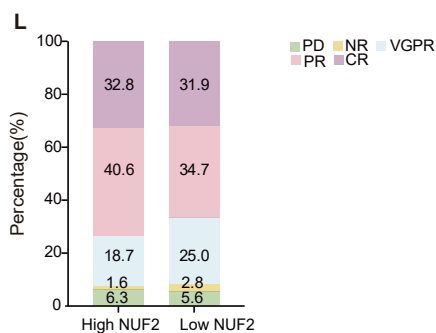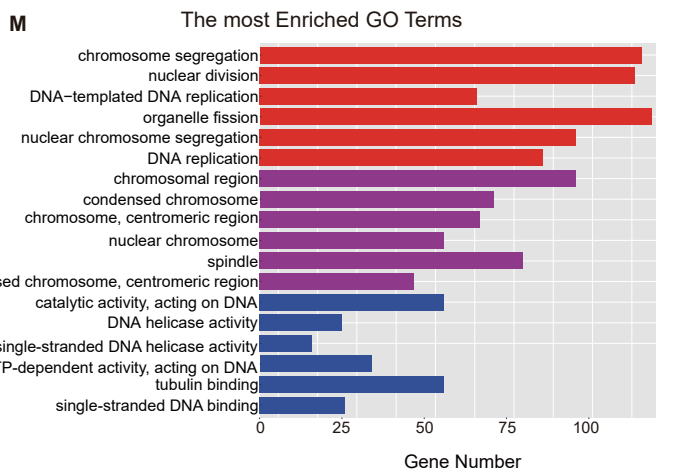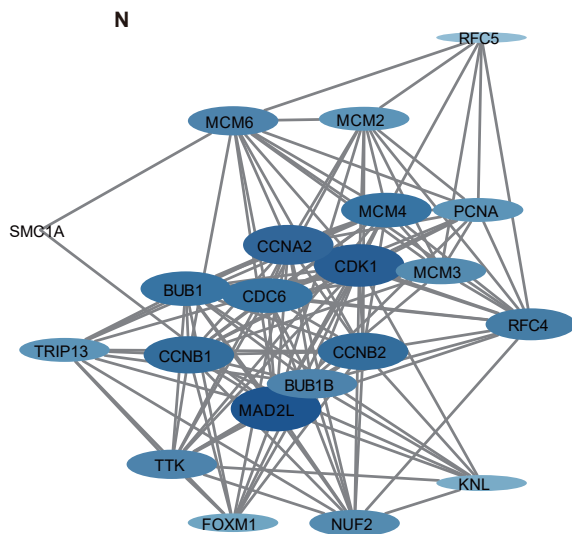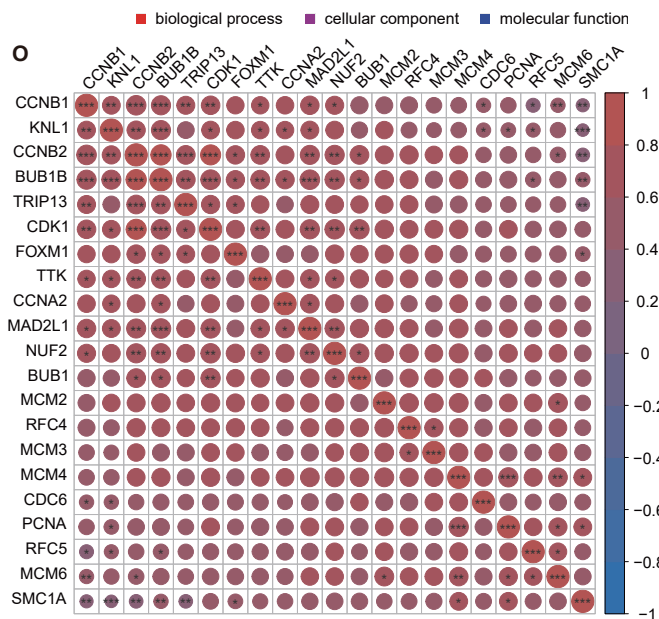

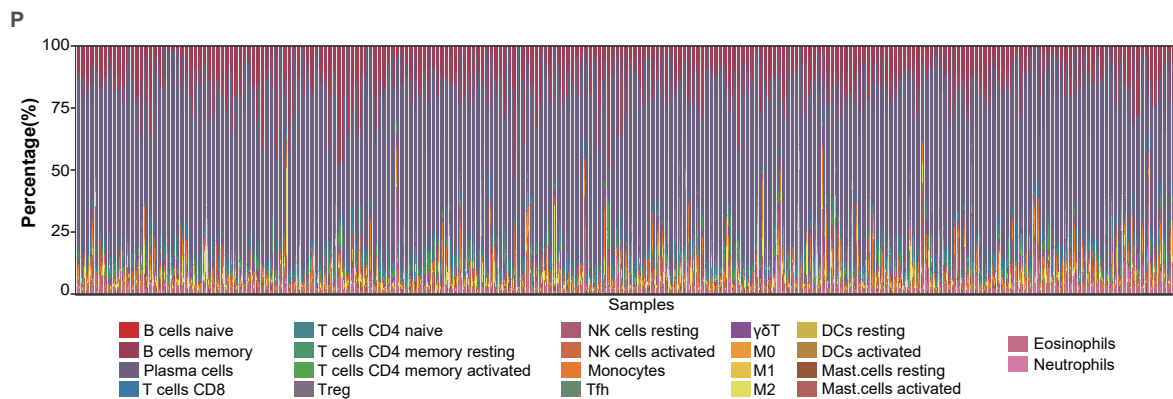

Supplement: Figure S1 — Identification and validation of the prognostic value of CRIP1 in multiple myeloma (MM). (A) Volcano plot of differentially expressed genes in MM patients with and without 1q gain/amp. (B) The Venn diagram illustrating the overlap between the up-regulated genes in Chr1q (+) patients and the genes located on Chr1q. (C) The Kaplan–Meier curves comparing the overall survival of MM patients grouped by NUF2 expression and 1q gain/amp. (D) Survival curves of event-free survival from dataset GSE24080. (E) Survival curves of overall survival from dataset GSE24080. (F) Multivariate Cox proportional hazards regression for event-free survival from dataset GSE24080. (G) Multivariate Cox proportional hazards regression for overall survival from dataset GSE24080. (H) A prognostic nomogram that integrated NUF2 expression and other prognostic factors for overall survival in MM from GSE24080. (I) A calibration curve at 1-, 3-, and 5-year. (J) The heatmap showing the main clinical characteristics and P values of MM patients grouped by high and low NUF2 expression. (K) The boxplots displaying the relations of NUF2 expression to main clinical parameters. (L) The relation of NUF2 expression to treatment response. (M) The GO functional enrichment analysis of the NUF2 co-expressed genes. (N) The top 20 genes of the cell cycle, cellular senescence, and DNA replication correlated with NUF2 in the protein–protein interaction network. (O) Correlation between NUF2 and main candidate genes enriched. The correlation coefficients were calculated by Pearson correlation analysis. ∗P ≤ 0.05, ∗∗P ≤ 0.01, ∗∗∗P ≤ 0.001. (P) The landscape of the composition of the 22 immune cells in each sample, and the samples are in ascending order of NUF2 expression along the x-axis. The P values in (C–E) were calculated by the Log-rank test, the P values in (F–H) were calculated by the autoReg R package, and the P value in (L) was calculated by the chi-square test. [file mmc2.pdf]
